# African Swine Fever Vaccinology: The Biological Challenges from Immunological Perspectives

**DOI:** 10.3390/v14092021

**Published:** 2022-09-13

**Authors:** James J. Zhu

**Affiliations:** US Department of Agriculture, Agricultural Research Service, Foreign Animal Disease Research Unit, Plum Island Animal Disease Center, Orient, NY 11957, USA; james.zhu@usda.gov

**Keywords:** African swine fever virus, ASFV, infectious virions, glycan shield, antigen surface density, apoptotic mimicry, virus receptors, virus neutralization, immune evasion, immune protection

## Abstract

African swine fever virus (ASFV), a nucleocytoplasmic large DNA virus (NCLDV), causes African swine fever (ASF), an acute hemorrhagic disease with mortality rates up to 100% in domestic pigs. ASF is currently epidemic or endemic in many countries and threatening the global swine industry. Extensive ASF vaccine research has been conducted since the 1920s. Like inactivated viruses of other NCLDVs, such as vaccinia virus, inactivated ASFV vaccine candidates did not induce protective immunity. However, inactivated lumpy skin disease virus (poxvirus) vaccines are protective in cattle. Unlike some experimental poxvirus subunit vaccines that induced protection, ASF subunit vaccine candidates implemented with various platforms containing several ASFV structural genes or proteins failed to protect pigs effectively. Only some live attenuated viruses (LAVs) are able to protect pigs with high degrees of efficacy. There are currently several LAV ASF vaccine candidates. Only one commercial LAV vaccine is approved for use in Vietnam. LAVs, as ASF vaccines, have not yet been widely tested. Reports thus far show that the onset and duration of protection induced by the LAVs are late and short, respectively, compared to LAV vaccines for other diseases. In this review, the biological challenges in the development of ASF vaccines, especially subunit platforms, are discussed from immunological perspectives based on several unusual ASFV characteristics shared with HIV and poxviruses. These characteristics, including multiple distinct infectious virions, extremely high glycosylation and low antigen surface density of envelope proteins, immune evasion, and possible apoptotic mimicry, could pose enormous challenges to the development of ASF vaccines, especially subunit platforms designed to induce humoral immunity.

## 1. Introduction

African swine fever virus (ASFV) is a nucleocytoplasmic large DNA virus (NCLDV) that infects wild boars and domestic pigs. The infection causes acute hemorrhagic disease, called African swine fever (ASF), with mortality rates up to 100% in pigs [1]. The outbreaks have been identified on all continents except for Australia and are considered one of the biggest threats to the swine industry. ASFV is quite complex, containing a large genome, encoding ~170 protein genes [2,3,4] and produces various infectious virions with sophisticated structures [5]. Proteomic analysis showed that mature virions contain a large number of viral (~70) and host (>20) proteins [6]. Several ASFV proteins are able to evade host immune response [7].

The NCLDVs belong to the Nucleocytoviricota phylum consisting of nine families. These families share certain genomic and virion characteristics, such as homologous genes involved in DNA repair, DNA replication, transcription, and translation, indicating the existence of a common viral ancestor [8]. Among NCLDVs, poxviruses, including the best thoroughly studied variola and vaccinia viruses, are the most genetically similar to ASFV [9]. Both poxviruses and ASFV infect mammalian hosts, have cell tropism for macrophages, and share several biological characteristics, e.g., multi-layer virion structure. However, there are major differences in virion components, the ranges of hosts, and cell tropism. Poxviruses have a broad range of mammalian hosts and cell tropism infecting epithelial cells, dendritic cells, monocytes/macrophages, B cells, and activated T cells [10], whereas ASFV primarily infects the macrophages of wild boars and pigs [11].

Recent approval of the ASFV-G-∆I177L live attenuated virus (LAV) platform manufactured by Navetco in Vietnam marks the first commercial ASF LAV. Research is still needed for the regulatory approval of these platforms. Outside of LAV, all other attempts were not shown to effectively protect pigs against highly virulent strains of ASFV [12,13]. Even when ASF vaccines become available in endemic countries, it is not expected that the outbreaks will end soon due to the presence of virus reservoirs in wild boars, including soft ticks and widely diverse ASFV existing in Africa. ASFV is also known to exhibit high infectivity and viral resistance to environmental conditions, further complicating the situation in endemic countries.

## 2. ASF Vaccine Research

ASF vaccine research has been conducted since the 1920s [14]. Several excellent review articles on ASF vaccine research have been published [12,13,14,15,16,17]. Many experimental ASFV vaccine platforms have been investigated, such as inactivated, subunit, and LAV vaccines. Like inactivated vaccinia virus vaccines, inactivated ASFV, even with adjuvant and high doses, failed to protect pigs [18,19]. However, inactivated lumpy skin disease virus (poxvirus) vaccines were protective in cattle [20,21]. In contrast to several subunit poxvirus vaccines, which induced immune protection [22,23,24,25,26], all subunit ASF vaccine candidates tested thus far, including recombinant viral proteins, DNA plasmids, and viral vectors containing several ASFV structural genes, did not induce sufficient immune protection in pigs [13,14]. Similar to poxvirus LAV vaccines, several attenuated ASFVs were shown to induce highly effective protection against ASFV challenge [2].

ASF LAV vaccine candidates have not been widely tested. Reports thus far show that the immune protection induced was different in terms of the onset and duration of protection in comparison to LAV vaccines for other diseases. E.g., classical swine fever [27] and smallpox [28,29] LAV vaccines were demonstrated to protect at 3 days after vaccination. LAV ASF vaccines have not been reported to induce protection shorter than two weeks after vaccination. One study showed that single intramuscular immunization with attenuated OURT88/3 or BeninΔMGF did not protect vaccinated pigs after challenge with virulent Benin 97/1 isolate at 130 days postimmunization [30]. This immune protection duration is much shorter than those reported for smallpox and classical swine fever LAVs [31,32]. Additionally, unlike poxvirus LAV, which induced excellent cross-protection, several experimental LAV ASF vaccines showed a lack of cross-protection against different serotypes based on hemadsorption inhibition assays and/or genotypes [14,33]. However, cross-protection has been reported for one experimental LAV, BA71ΔCD2 [34].

## 3. Biological Challenges

The difficulties involved in ASF vaccine development are mainly due to the complexity of the virus and lack of knowledge regarding the mechanisms of immune protection and protective antigens [17,35,36,37,38,39]. This review focuses on several ASFV characteristics published at the time of this review and infers their potential impacts on vaccine development from immunological perspectives based on the knowledge learned from studies of other viruses.

### 3.1. Extracellular and Intracellular Virions Are Infectious and Abundant

Similar to poxviruses [40], both intracellular and extracellular ASFV are infectious [41,42,43]. Infectious intracellular virions of ASFV Hinde isolate were present in cultured porcine kidney cells at 2 h and from 24 to 96 h post infection (hpi) [41]. There were ~10-times more intracellular virions than extracellular virions at 24 hpi for this virus strain. It was reported that ~25% of total virions were extracellular virions at 48 hpi for a genotype I isolate, BA71V [44]. In our experiment, infectious intracellular virions of ASFV Georgia 2007 were present in adherent pig PBMC at 10, 11, and 12 hpi. Extracellular virions appeared at ~13 hpi and consisted of ~40% of total virions at 15 hpi [43]. These results showed that the percentage of extracellular ASFV virions was significantly higher than those of extracellular poxvirus virions, which are mostly less than 1% [45]. In vaccinia virus, outer envelope proteins A33 and A34 are C-type lectins [46]. Deletion of either gene increased the output of extracellular virions from infected cells [47,48]. There is only one ASFV C-type lectin gene, EP153R; however, a similar mechanism for ASFV has not been tested.

Extracellular poxvirus virions are further divided into two biologically distinct virions: extracellular enveloped virions that bud directly from the plasma membrane and cell-associated enveloped virions (CEVs) that bud through plasma membrane by propulsion of actin tails and remain attached to or are released from the cell membrane [49,50]. CEVs have been reported in ASFV [51,52]. Intracellular ASFVs include intracellular mature (IMV) and immature (IV) virions [53]. Antibodies against surface proteins of intracellular virions, such as capsid protein p72 and inner membrane proteins p30/32 and p54, showed neutralizing activity and partial protection against ASFV infection [54,55,56]. Both extracellular and intracellular virions probably play a role in ASF pathogenesis in pigs. Multiple infectious ASFV virions could be a challenge for vaccine development, which might explain, in part, why a vaccine trial using intracellular virion antigens (p30, p54 and p72) did not show protection against virus challenge, even in the presence of neutralizing ASFV antibodies in vaccinated pigs [57].

### 3.2. CD2v and C-type Lectin Are Extremely Glycosylated

Most viral envelope proteins are glycosylated (N-linked and/or O-linked) in the ER and the Golgi complex [58]. CD2v is the only known outer membrane protein based on its hemadsorption activity in infected cells [59] and detection in mature virus particles [6,53]. The location of C-type lectin on the ASFV outer membrane remains to be determined, which was not detected in mass spectrometric analysis of extracellular ASFV virions [6]. Given that both C-type lectin glycoproteins (A33 and A34) of vaccinia virus are located on the outer envelope [46], especially in CEV [50], ASFV C-type lectin may also be located on the outer envelope but at a level below detection.

ASFV CD2v and C-type lectin were found to be extremely glycosylated [60,61]. Analysis with bioinformatic programs detected 14 potential N-linked glycosylation sites in the ectodomain (~190 amino acids) of CD2v in contrast to only two sites in pig CD2. Likewise, there are eight N-linked glycosylation sites in the ectodomain (103 amino acids) of C-type lectin. CD2v and C-type lectin have approximately one N-linked glycosylation site per 13 amino acids, which is much higher than other ASFV structural proteins and extracellular outer membrane proteins of poxviruses, e.g., one site per ~45 amino acids for hemagglutinin A56R (the highest among five outer membrane proteins of vaccinia virus). Glycans consist of approximately 55% and 65% of CD2v [60] and C-type lectin [61] molecular weights (MW), respectively, based on the MW observed and calculated. Experimental results suggest that CD2v and/or C-type lectin might be protective antigens [62,63]. Additional deletion of CD2v and C-type lectin genes in attenuated ASFVs reduced or eliminated the ability of the LAVs to protect pigs [64,65].

Glycosylation plays a key role in enveloped virus pathobiology in terms of virus release, cell infection, and immune evasion [58]. Glycans are poorly immunogenic because of (1) inherently weak carbohydrate–protein binding affinities, (2) heterogeneity of glycan structures in the same protein backbones, and (3) tolerogenic self-like glycan structures [66]. These self-like glycans can serve as immunological silencers of viral proteins to avoid recognition by immune cells. N-linked glycans of HIV Env spike consist of approximately 50% of the MW. The negative impact of such a high degree of glycosylation on the immune response is named ‘glycan shield’ [66,67]. Additionally, peptides containing glycans might be difficult for MHC to load due to size restriction in the binding groove [68,69]. It is a significant challenge to develop vaccines that induce neutralizing antibodies targeting highly glycosylated HIV spike protein. The challenge could be similar to vaccines for inducing neutralizing antibodies to ASFV CD2v and C-lectin-like proteins. Anti-hemadsorption antibodies were detected at low titers in most pigs infected with an attenuated virus and undetectable in those immunized with purified CD2v protein [70], indicating that CD2v is a poor immunogen. Our unpublished data show similar results with high titers of CD2v antibodies observed in vaccinated pigs only after virulent ASFV challenge.

### 3.3. Estimated Surface Densities of Most Envelope Proteins Are Very Low

In a study using mass spectrometry (MS) [6], CD2v was detected in two of three purified ASFV extracellular virion samples, but C-type lectin was not detected at all. The abundance (the weight percentages of the total virion protein mass) of CD2v was nearly the lowest among the detected ASFV structural proteins in the study. In contrast, all five outer envelope proteins of vaccinia virus were detected in a similar study [71]. The surface density of antigens plays a key role in the antigenicity and immunogenicity. HIV has the lowest surface density (0.01 per 100 nm^2^) of spikes among enveloped viruses, which is one of the immune evasion mechanisms of HIV to reduce antibody production and to avoid neutralization by antibodies [72,73]. The distance between two extended bivalent IgG Fab binding sites is ~15 nm [72]. Therefore, for avidity to work, Fab_2_-IgG antibodies need a density of approximately two epitopes within an area of 100 nm^2^. It was reported that the binding of low-affinity antibodies to HIV particle was more affected by low antigen density [74].

Reported MS results, including the abundance and molecular weights of structural proteins in ASFV mature particle (extracellular virions) [6], were used to estimate the molecular number and surface density of structural proteins on the capsid, inner and outer membranes, and core shell. The estimated surface density of CD2v is the lowest at 0.03 protein per 100 nm^2^ among the surface proteins (Table 1). Three neutralizing antibody-inducing inner membrane proteins, p12, p30/p32, and p54, have a density at 0.94, 0.13, and 0.33, respectively. An additional two inner membrane proteins, p17 and p22, have a density of 8.7 and 2.1 proteins per 100 nm^2^, respectively, but the neutralizing activity of their antibodies have not yet been demonstrated. The major capsid protein (p72) has a density at 5.3 proteins per 100 nm^2^ and other capsid proteins are less than 1. All core shell proteins (p8, p14, p34, and p35) have 3.8 or more proteins per 100 nm^2^. These results indicated that the densities of neutralizing antibody-inducing outer and inner envelope proteins were well below one protein per 100 nm^2^. The low surface densities of CD2v, p30, p54, and possibly C-type lectin would reduce not only the avidity of the antibodies to the virions (antigenicity) but also the immunogenicity of LAVs. However, it must be pointed out that these estimations could be significantly biased by several factors such as the number and accessibility of tryptic sites in the proteins, protein solubility, modifications, etc. (personal communication with Dr. Germán Andrés).

Low surface density of CD2v on extracellular virions could, in part, be due to the majority of expressed CD2v localization within infected cells rather than on the cell surface [60]. Some CD2v proteins are cleaved into a large (63 kDa) highly glycosylated N-terminal luminal fragment (the ectodomain) and a small (26 kDa) C-terminal non-glycosylated membrane-attached fragment in ASFV-infected cells [60]. Another study indirectly supports the low surface density of CD2v on virus particles, showing that hyperimmune sera prepared using recombinant CD2v protein, but not convalescent sera from pigs that were recovered from ASF, detected CD2v in virus particles by Western blotting [54]. For ASFV C-type lectin, its binding to MHC-I proteins and blocking MHC-I exocytosis [78] could reduce its expression on the cytoplasm membrane.

### 3.4. Naïve Sera Enhance ASFV Infection

We found that naïve sera can enhance ASFV infection compared to the culture medium. It was further shown that extracellular but not intracellular virions suspended in naïve sera were more infectious than those in culture medium [43], suggesting that certain components in naïve sera can increase ASFV infectivity. Serum-enhanced virus infection in macrophages has been reported for HIV [79]. The serum components involved in facilitating infection of several enveloped viruses, such as dengue virus, Ebola virus, HIV, and poxviruses, have been identified to be phosphatidylserine (PtdSer) binding serum proteins (Protein S, Gas6, and Mer), which act as bridging molecules for TAM receptors (Tyro3, Axl and Mer) expressed on macrophages during infection via clathrin-mediated endocytosis [80,81,82].

It is well known that apoptotic cells expose PtdSer on the outer leaflets of their plasma membrane due to inactivation of flippases by activated caspase 3. PtdSer serves as an “eat me” signal for macrophages to engulf dying cells [83,84]. Virus infection activates caspase 3 to expose PtdSer on the surface of infected cells. PtdSer then becomes an envelope component during virus budding. Infection mediated by envelope PtdSer and cell PtdSer receptors is an example of apoptotic mimicry in viruses [85]. This mechanism was also reported for porcine reproductive and respiratory syndrome virus that also targets pig macrophages [86]. Interestingly, serum proteins enhanced infection of extracellular, but not intracellular, virions of vaccinia virus [87], similar to the results observed for ASFV in our study [43].

ASFV induces apoptosis in infected macrophages [88] and in adapting cells [89] by activating caspase 3 [44]. Inhibition of caspase 3 activity during early ASFV infection blocked the production of the extracellular virions but not total virions [44]. Interestingly, a similar phenomenon was observed when inhibition of PtdSer synthesis, trafficking, or scrambling reduced the release of Ebola virus from infected cells [90,91]. ASFV acquires an outer envelope by budding through the plasma membrane [5]. These findings suggest that exposing PtdSer on the outer leaflets of the plasma membrane of infected cells is required for ASFV budding. These observations together with serum-enhanced infectivity of extracellular ASFV virions strongly support the involvement of PtdSer in ASFV infection, similar to other enveloped viruses.

Our results and others showed that monocyte-derived macrophages were ~5-times more susceptible to ASFV infection than monocytes in vitro [43,92,93,94]. Macrophages are the primary cells clearing apoptotic cells and have a higher capacity of phagocytosing apoptotic cells than monocytes [84,95,96]. The hypothesis of PtdSer on the ASFV envelope and its involvement in ASFV infection could explain, in part, why ASFV primarily infects macrophages and why CD2v or C-type lectin-knockout ASFV remains infectious. The role of PtdSer in ASFV infection, if it exists, could be another major obstacle for ASF vaccine development (more discussion later).

### 3.5. Virus Receptors Are Unknown but Likely Numerous

ASFV primarily infects macrophages in pigs, suggesting restricted expression of macrophage-specific virus receptors as the main mechanism of virus entry [13]. ASFV infects pig macrophages via both clathrin-mediated endocytosis and macropinocytosis [97,98]. It has been demonstrated that monocytic cells became susceptible to ASFV infection when SWC9 (possibly CD80) expression was upregulated [93]. Anti-CD163 antibody inhibited ASFV infection and binding to cells [99]; however, CD163 expression alone was not enough for ASFV infection [100]. CD163-knockout pigs were not resistant to the infection [101]. These results suggested that other receptors were also involved in ASFV infection [102]. CD2v binds to host CD58 as host CD2 does [103], which could be one of the ASFV receptors. CD58 is highly expressed on antigen-presenting cells, including macrophages [104].

CD2v and C-type lectin are heavily glycosylated, as previously described. The N-linked glycans in virus spike proteins are important for viral infectivity [105,106]. Therefore, the N-linked glycans of CD2v and possibly C-type lectin could serve as ligands for host glycan binding proteins (GBPs), such as DC-SIGN and C-type lectins. Mice macrophages express high levels of GBPs on their surface [107]. Nevertheless, CD2v [108,109] or C-type lectin alone is not essential for ASFV replication [61], indicating that there could be more receptors involved in ASFV infection.

As previously mentioned, PtdSer receptors could serve as ASFV receptors for cell infection, like several other enveloped viruses, via apoptotic mimicry [81,82]. Additionally, host membrane adhesive proteins, e.g., CD54 and ITGα4β7 on the HIV envelope, increased virus infectivity [110]. Several host membrane proteins, such as CD9, ITGα3β1, and ITGαVβ1, were also detected in ASFV extracellular virions [6] (Alejo et al., 2018), which could bind to their respective receptors to enhance ASFV infectivity. The high expression of PtdSer receptors and GBPs in macrophages could explain, at least in part, why macrophages are the primary target cells of ASFV. These ASFV receptor candidates are summarized in Table 2. Possible immunity-mediated enhancement of ASFV infection and disease was observed in some vaccine-challenge studies [13] (Gaudreault and Richt, 2019); however, Fc receptors appear not to be involved in ASFV infection [111]. Therefore, the involvement of poorly or non-immunogenic PtdSer and host proteins in ASFV infection could be another major factor underlying the difficulties in ASF vaccine development.

### 3.6. Antibodies Cannot Completely Neutralize ASFV

Virus neutralization by antibodies is one of the major protective mechanisms against viral infections. However, ASFV-specific antibodies showed a partial neutralization effect [57,112,113,114,115,116,117]. Even in the presence of a high concentration of antibodies, such as monoclonal antibodies or hyperimmune sera, from pigs infected with an attenuated virus and/or survived/protected after challenge with a virulent wildtype virus, approximately 5 to 20% of the virions remain non-neutralized [43,112,114,116,117,118]. Antibodies to p72 and p54 inhibited virus attachment, whereas antibodies of ASFV p30 inhibited virus internalization [116]. Among three pigs tested in an experiment, the serum from a pig inoculated with three high doses of cells infected with a CD2v-expressing baculovirus showed ASFV neutralizing activity at 5 days after injection and all three vaccinated pigs survived after challenge and had neutralizing antibodies [54]. These results indicated that CD2v, p30, p54, and p72 are protective antigens. Although recombinant p12 (an inner and possibly also an outer membrane protein) can inhibit ASFV infection, antibodies to ASFV p12 showed poor or no neutralizing activity and pigs vaccinated with recombinant p12 were not protected [116,119,120]. The neutralizing activity of antibodies to C-type lectin, p17, and p22 has not been reported.

Vaccinia virus extracellular virions are more resistant to antibody neutralization than intracellular virions [121]. Both extracellular and intracellular ASFV virions could not be completely neutralized by hyperimmune sera [43]. The mechanisms involved in incomplete ASFV neutralization are not yet clear. It has been demonstrated that the preincubation of ASFV with non-neutralizing sera inhibited the effect of ASFV-neutralizing sera [117], which, in part, is consistent with the naïve serum-enhancing effect on ASFV infectivity. More interestingly, removal of phosphatidylinositol (PtdIns) from ASFV particles decreased neutralization efficacy, whereas adding PtdIns increased neutralization [122]. Both PtdSer and PtdIns are anionic phospholipids. Uneven distribution of these phospholipids between the outer and inner leaflets induces membrane curvature [123,124], which might be required for ASFV budding as discussed earlier. It may be speculated that the presence of excessive PtdIns interferes with the binding between PtdSer and PtdSer receptors, therefore, blocking apoptotic mimicry. In this scenario, ASFV may rely solely on its own proteins for cell infection, increasing its susceptibility to antibody neutralization. The combination of high glycosylation, low antigen surface density, apoptotic mimicry, and the presence of host membrane proteins on the outer envelope could contribute to ASFV incomplete neutralization.

### 3.7. Viral Proteins Control Apoptosis and/or Inhibit MHC-I Expression

ASFV infection triggers apoptosis in infected cells [44,88,89]. There are several ASFV proteins, such as CD2v, MGF360, MGF505, and E183L, that have pro-apoptotic activity [102,125,126]. On the other hand, ASFV also expresses several anti-apoptotic proteins, such as A179L, A224L, EP153R, and DP71L [127,128]. Additionally, pEP153R inhibits the expression of MHC class I molecules on the cytoplasmic membrane of infected cells [78]. Inhibition of apoptosis and MHC Class I expression by ASFV proteins could undermine the cytotoxicity mediated by antibodies, NK, and/or antigen-specific T cells, which could, in turn, undermine the efficacy of vaccine-induced immunity.

### 3.8. Protective Immune Mechanisms Are Not Well Understood

Both humoral and cell-mediated mechanisms have been investigated for their contribution to protective immunity. The role of antibodies in protection against ASFV infection was well described in a review publication [129]. Similar to the protective effect of passively transferred antibodies of vaccinia virus in mice [130,131], the antibody protective activity was convincingly demonstrated in pigs with passive transfer of colostrum or serum antibodies from convalescent pigs [132,133,134]. Although ASFV cannot be completely neutralized in in vitro assays with hyperimmune sera from convalescent or challenged and protected pigs, as discussed earlier, antibodies can kill ASFV-infected cells via complement-mediated cytotoxicity (CDC) [135] and antibody-dependent cellular cytotoxicity (ADCC) [136] in vitro. It may also be confidently speculated that ASFV could also be cleared by antibody-dependent cellular phagocytosis (ADCP), though it has not been reported. Despite these anti-ASFV activities of antibodies, subunit vaccines containing several potentially protective antigens of both extracellular and intracellular virions, such as CD2v, p72, p30, and p54, failed to protect pigs though antigen-specific antibodies, though some neutralizing activities were detected in vaccinated pigs [118,137].

Cytotoxicity mediated by cytotoxic lymphocytes, such as NK and CD8+ T cells, has been demonstrated with in vitro ^51^Cr release assays [138,139,140,141]. Immune protection against ASFV was associated with cytotoxicity in CD8+ T cells [34,142] or increased NK activity [143] (Leitão et al., 2001). Pigs infected with an attenuated ASFV (OUR/T88/3) were no longer protected against virulent ASFV challenge after depletion of CD8+ lymphocytes [144]. Pigs immunized with plasmid DNA containing ASFV antigen genes and designed to activate only T cells were partially protected and showed an increased number of CD8+ cells in the blood at Day 3 post infection compared to control pigs [145]. Cross-protection induced by attenuated ASFV was correlated with cell-mediated immunity-associated parameters measured with IFN-γ ELISPOT, lymphocyte proliferation, and T cell epitope assays [146,147]. T effector cells induced by MHC epitopes of ASFV CD2v, C-type lectin, p30, pp62, and p72 were detected in infected, vaccinated, and/or protected pigs [148,149]. Immune recall response in pigs vaccinated with a cocktail containing multiple Ad5-vectored ASFV genes was detected with IFN-γ ELISPOT assays, but these pigs were not protected [150]. More interestingly, ASFV T cell epitopes were identified by systemic screening using IFN-γ ELISPOT assay, and Ad5 vectors inserted with all genes containing dominant T cell epitopes reduced viremia but did not prevent vaccinated pigs from severe disease [151]. Therefore, efficient protection in pigs by cell-mediated immunity alone remains to be a challenge. After all, cell-mediated cytotoxicity that prevents or reduces the release of infectious virions from ASFV-infected cells has not been demonstrated.

## 4. Conclusions

Although the protective mechanisms induced by live attenuated ASFVs are not clear, experimental results strongly indicate that both humoral and cell-mediated immunities contribute to the protection. Antibodies alone can provide highly effective protection in pigs via passive transfer and in virus neutralization assays. Antibodies together with other immune factors have anti-ASFV activity in CDC, ADCC, and possibly ADCP assays. In contrast, similar in vivo protection and in vitro inhibition of ASFV production by cell-mediated immunity alone has not been demonstrated. Cell-mediated immunity-associated parameters were detected with in vitro cytotoxicity or IFN-γ ELISPOT assays in samples collected from vaccinated/protected pigs. Given the evident protective effects of antibodies and that most of, if not all, the protective antigens involved can be inferred from the experimental results and/or knowledge in ASFV, why did subunit vaccines designed based on these antigens to induce antibody production fail to protect pigs? The complexity of ASFV may hinder the efficacy of the subunit vaccines; however, subunit vaccines containing the envelope proteins are effective for poxviruses whose complexities are comparable to ASFV. The ineffectiveness of ASF subunit vaccines is most likely due to several unusual characteristics discussed in this review, including (1) multiple biologically distinct infectious virions; (2) extreme glycosylation of CD2v and C-type lectin; (3) low surface densities of most envelope proteins; and (4) possible host membrane proteins and PtdSer on the envelope facilitating ASFV infection. Similar characteristics in HIV are well known to be enormous obstacles in AIDS vaccine development. Extreme glycosylation and/or low surface density of ASFV envelope proteins may not only reduce the immunogenicity of the protective antigens but also decrease the antigenicity of the virions. These unusual features could pose enormous challenges for ASF vaccine development, especially subunit platforms. The challenges might be too great for today’s subunit vaccine technologies to overcome. Additionally, several ASFV proteins inhibit apoptosis and/or MHC Class I expression, which could undermine the effectiveness of vaccine-induced cytotoxicity in killing infected cells; however, why T cell epitope-based subunit ASV vaccines did not induce sufficient protection in pigs remains to be investigated.

## Figures and Tables

**Table 1:** Estimated number of proteins per virion (Protein#) and protein density (Protein#/100 nm^2^) of known viral proteins on ASFV outer membrane (OuterM), capsid, inner membrane (InnerM), and core shell based on reported protein abundance (%) in ASFV particles measured with mass spectroscopy, molecular weights (MW), and virion surface areas.

Location	Protein	Gene	Abundance ^1^	MW ^1^	Protein# ^2^	Density ^3^
OuterM	CD2v	EP402R	0.06	46.5	82	0.03
	C-type lectin	EP153R	not detected	18.4	unknown	unknown
Capsid	p72	B646L	9.55	73.6	8280	5.26
	p49	B438L	0.93	49.6	1196	0.76
	pM1249L	M1249L	2.07	145.3	909	0.58
	penton protein	H240R	0.38	27.7	875	0.56
	pE120L	E120L	0.08	13.6	375	0.24
InnerM	p17	D117L	2.12	13.2	10249	8.67
	p22	KP177R	0.79	20.7	2435	2.06
	Fusion protein	E248R	0.91	27.7	2096	1.77
	p12	O61R	0.12	6.9	1110	0.94
	Fusion protein	E199L	0.23	22.7	647	0.55
	p54	E183L	0.12	19.9	385	0.33
	pH108R	H108R	0.05	12.5	255	0.22
	p30/p32	CP204L	0.13	23.6	352	0.13
Core shell	p34	CP2475L	19.43	36.6	33876	38.2
	p14	CP2475L	5.46	17.9	19465	22.0
	p35	CP530R	5.04	35.2	9137	10.3
	p8	CP530R	0.41	7.8	3354	3.78
Nucleoid	Histone-like	A104L	4.64	11.6	25525	N/A
	DNA binding	K78R	0.85	8.4	6457	N/A

^1^ The data were obtained with permission from a published mass spectroscopy study by Alejo et al. (2018) [6]. ^2^ The number of structural proteins (N_x_) was estimated with the equation: N_x_ = N_p72_ × (A_p72_ ÷ A_x_) × (MW_p72_ ÷ MW_x_), where A and MW are the abundance (%) measured with mass spectrometry and molecular weight, respectively; N_p72_ is the number of p72 proteins (8280) per ASFV virion reported by Andrés et al. (2020), Liu et al. (2019) and Wang et al. (2019) [75,76,77]. ^3^ The estimated density (the number of a protein per 100 nm^2^) is equal to N_x_ divided by S and multiplied by 100, where surface areas (S) were calculated using the formula (S = 4πr^2^) for speres instead of icosahedrons as approximation and r is the radius of the virions based with permission on the report by Andrés et al. (2020) [75].

**Table 2:** Virus receptor candidates for ASFV extracellular (outer membrane) and intracellular (capsid and inner membrane/innerM) virions based on the potential binding between virus components and host receptors.

Virus		Host	
Virion	Component	Serum Protein	ASFV Receptor Candidate
Extra-cellular	CD2v		CD58 ^1^, CD15, CD48, and CD59
	C-type lectin (?)		MHC Class I ^2^, Glycans of CD163, CD107a ^3^
	N-linked glycans		GBPs: DC-SIGN, C-type lectins, etc ^4^
	phosphatidylserine (PtdSer) ^5^		CD36, CD300, TIMD4, BAI-1, stabillin
		MFG-E8	ITGαVβ3
		C1q	C1qR, CR1
		Gas6, Protein S	AXL receptor, MER, TYRO3
	ITGα3β1 ^6^		CD9, CD36, CD46, CD82, CD151
	ITGαVβ1 ^6^		Receptors with RGD motif
	CD9 ^6^		CD29, CD46, CD49c, CD89, CD117
Intra-cellular	Capsid proteins		unknown
	InnerM proteins		unknown

^1^ CD2v binding to CD58 was reported by Chaulagain et al. (2021) [103]. ^2^ C type lectin binding to MHC- Class I molecules was reported by Hurtado et al. (2011) [78]. ^3^ CD163 association with ASFV infection was reported by Sánchez-Torres et al. (2003) [99]. ^4^ Glycans on CD2v and C-type lectin and glycan binding proteins (GBPs) are inferred based on Park et al. (2020) [107]. ^5^ PtdSer receptors are based on a review article by Amara and Mercer (2015) [85]. ^6^ Host proteins on ASFV virions are reported by Alejo et al. (2018) [6] and their binding proteins are according to the NCBI Gene database.

## Data Availability

Not applicable.
